# A Modern Clinical Approach of the Traditional Korean Saam Acupuncture

**DOI:** 10.1155/2015/703439

**Published:** 2015-10-11

**Authors:** Manyong Park, Sungchul Kim

**Affiliations:** ^1^Wonkwang University Korean Medical Hospital, Department of Acupuncture & Moxibustion, Gwangju, Republic of Korea; ^2^ALS Center of Wonkwang University Korean Medical Hospital, Gwangju, Republic of Korea

## Abstract

Saam acupuncture is one of the original therapeutic modalities representing traditional Korean medicine. It was originally described in a manuscript that is estimated to be published at some point between 1644 and 1742, in the middle of the Cho Sun dynasty, by a Korean Buddhist monk whose name is unknown. The principle of combining five shu points is based on the theory of Nan-jing. The treatment and diagnosis concepts in Saam acupuncture were mainly influenced by Dongeuibogam and Chimgoogyeong-heombang. The basic characteristic of combining five shu points in Saam acupuncture is the selection of the tonification and sedation points along the self-meridian and other meridians based on creation and governor relationships. Saam acupuncture clinical studies have mainly focused on musculoskeletal pain and autonomic nervous system regulation. From a neurophysiological perspective, Saam acupuncture, which involves five shu points as the main treatment aspect, has the advantage of increasing parasympathetic nerve activation and adjusting the balance of the autonomic nervous system. Inserting a needle into the skin layer while considering the respiratory phase and stimulating the needle gently and lightly could maximize the effect of Saam acupuncture. The specific Saam acupuncture prescribed should be identified on the basis of the neurobiological perspective.

## 1. Introduction

Saam acupuncture, which is one of the original therapeutic modalities representing traditional Korean medicine, is a unique treatment method that has a different origin than the modalities from China and Japan. The basic characteristic of combining five shu points in Saam acupuncture is the selection of the tonification and sedation points along the self-meridian and other meridians based on creation and governor relationships. In China, five element acupuncture, tonification, and sedation points along only the self-meridian are selected. Japanese meridian therapy added source point, connecting point, cleft point, alarm point, and transport point on the basis of Korea Saam acupuncture conception of the combined five shu points [1].

Saam acupuncture is based on the traditional concepts of yin-yang, five elements, ZangFu (viscera and bowels), qi, and meridians. Saam acupuncture treatment cannot be separated from these viewpoints. In particular, it involves the application of five shu points according to the creation and control cycles of the five-element theory. Therefore, the combination of acupoints in Saam is easier to understand from the perspective of traditional medicine.

The meridian is divided into three parts: the arm or foot, three yin and yang, and six ZangFu parts. A total of 24 deficiency and excess symptoms, with 24 coldness and fire symptoms, exist across the 12 meridians, but the diagnostic criteria related to these symptoms are too ambiguous for selecting a correct meridian. Except for the regular 48-treatment protocol, the treatment strategies are largely variable. Efforts were made to produce Saam treatments that are more effective by including other acupoints, with the main points firmly based on the regular pattern [2]. However, because the explanation regarding acupoint selection is very brief or elided, applying Saam acupuncture in the clinic is difficult.

Various Korean scholars have suggested different methods for applying Saam acupuncture in the clinic. Lee [3] proposed a diagnostic system by comparing pulse examination, whereas Kim [4] proposed a symptom-based diagnosis. Cho [5] established visceral pattern identification for providing easy access to Saam acupuncture by analyzing Neijing (Internal Classic), Nan-jing (Classic of Difficult Issues), and Yixuerumen (Introduction to Medicine). Moreover, Kwon [6] studied constitutional acupuncture, and Kim [7] reviewed the mind-based aspect of Saam acupuncture.

However, a description of the mechanism of acupuncture to both clinicians and patients in mainstream medicine by using the contemporary concepts of neurobiology would be helpful for incorporating Saam acupuncture into mainstream medicine [8]. An important difference between traditional Korean medicine and the Western medical approach is the diagnostic process. In traditional Korean medicine, the diagnosis is made by using the principles of syndrome differentiation, which could be in accordance with the state of the ZangFu or qi or with the doctrine of the meridians [9]. This diagnostic process does not match the patterns in Western medicine.

In this paper, several neurobiological mechanisms of Saam acupuncture treatment are presented from the perspective of the clinician. Saam acupuncture clinical trials are also discussed. Further, the historical background of Saam acupuncture and the basic principle of combining acupoints are briefly reviewed.

## 2. The Historical Background of Saam Acupuncture

The original manuscript that described Saam acupuncture is estimated to have been published between 1644 and 1742, in the middle of the Cho Sun dynasty, by a Korean Buddhist monk whose name is unknown. Because Chimgoogyeong-heombang (Experiential Prescriptions of Acupuncture and Moxibustion), which was published in 1644, is quoted in the manuscript of Saam acupuncture, the publication period of the Saam acupuncture manuscript can be estimated to be after 1644 [10]. After 1742, Gy-san's clinical experience was added to the original manuscript of Saam acupuncture, that is, Gyeongjeyogyeol (Essential Rhymes on Acupuncture and Moxibustion by Master Saam). This is the oldest existing manuscript associated with Saam acupuncture. The physiology, pathology, and disease identification sections of Saam were handed down to its disciples, but the clinical experience section of Gy-san coexists in the currently published Saam acupuncture-related books [11].

Viscera/bowel-based acupuncture was developed in Chimgoogyeong-heombang (Experiential Prescriptions of Acupuncture and Moxibustion), with integration of the meridian/exterior theory with the viscera manifestation theory, which in turn provided various methods for viscera diagnosis. Viscera identification became the basis of Saam acupuncture treatment [12].

The Dongeuibogam (Treasured Mirror of Eastern Medicine) contains the following phrase: “One acupuncture needle in all the diseases, up to within four acupuncture needles, acupuncture on the whole body is not a good idea.” This phrase represents the characteristics of the traditional Korean acupuncture method. In this context, Saam acupuncture treated the disease within four acupuncture points. Further, an acupuncture method based on the five element principles is presented in the acupuncture section of the Dongeuibogam. The idea of treating a disease derived from viscera/bowel dysfunction by using five shu points influenced Saam acupuncture [1].

## 3. The Basic Theory of Combining Five Shu Points in Saam Acupuncture

The following phrase is taken from the 69th chapter of Nan-jing: “In the case of depletion, fill the respective mother, in the case of repletion, drain the respective child, one must fill the first and then drain afterward.” Gao-Wu, of the Ming Dynasty of China (1519 A.D.), was the first acupuncturist to describe the use of tonification and sedation points along the self-meridian by using the five shu points according to the 69th issue of Nan-Jing. Zhang Shi Xian advocated the use of five shu points in other meridians in Jiao Zheng Tu Zhu Nan Jing (Illustrated note of Classic of Difficult Issues) [11]. On the basis of Gao-Wu and Zhang Shi Xian treatment, Saam added the role of the governor. This notion originated from the 50th and 75th chapters of Nan-Jing.

In Saam acupuncture, the relationship with the governor is important, as is the relationship between the mother and son. The governor is sedated under the condition of deficiency and is tonified under the condition of excess [13].

The basic rules are those of the creation and governor relationships. In the case of insufficiency of any meridian, the mother points of its mother and its own meridians should be tonified and the governor points of its governor and its own meridians should be sedated. For example, the following is applied if the lung meridian is diagnosed as deficient: earth tonification, lung meridian-earth point LU9 and spleen meridian-earth point SP3 and fire sedation, lung meridian-fire point LU10, and heart meridian-fire point HT8 (Table 1). The other meridians follow the same rule, as described above.

In the case of the excess of any meridians, the governor points of its governor and its own meridians should be tonified and the son points of its son and its own meridians should be sedated. For example, the following is applied if the lung meridian is diagnosed to be excessive: fire tonification, lung meridian-fire point LU10, and heart meridian-fire point HT8 and water sedation, lung meridian-water point LU5, and kidney meridian-water point KI10 (Table 2).

Another simple but rarely used one is coldness-fire acupuncture treatment derived from the deficiency-excess acupuncture treatment. For cold symptoms, the fire points of its own and the fire meridians are tonified and the Water points of its own and the water meridians are sedated. For heat symptoms, the water points of its own and the water meridians are tonified and the fire points of its own and the fire meridians are sedated (Table 2).

Among the clinical case studies of Saam, 85% used either a tonification or a sedation formula, whereas 15% used variations of the tonification and the sedation formulae [13]. Saam treatment primarily focuses on the viewpoints of deficiency-excess symptoms rather than cold-heat symptoms [1]. The 240 acupuncture treatments described in the Gyeongjeyogyeol consist of 100 regular forms on the basis of the 69th issue of Nan-Jing, 140 irregular forms on the basis of the 73rd and 75th issues, and various special acupuncture points, which include the source, connecting, accumulation, alarm, and back-transporting points [14].

## 4. Saam Acupuncture Clinical Research

To review clinical articles on Saam acupuncture, we used the following six databases: “Korean Studies Information Service system (KISS)”, “National Discovery for Science Leaders (NDSL),” “Research Information Sharing Service (RISS),” “Oriental Medicine Advanced Searching Integrated System (OASIS),” PUBMED, and Google Scholar. The key words were “Saam” and “Saam acupuncture.” The retrieved papers were screened so that only articles related to clinical research were retained. We selected 28 case studies and 17 clinical studies.

### 4.1. Saam Acupuncture Case Studies

Saam acupuncture has been applied for several diseases, and these are summarized in Table 3. However, the number of cases is low, and various therapeutic modalities were combined with Saam acupuncture except for some cases. Therefore, recognizing the effect that can be attributed to Saam acupuncture is difficult. Notably, treatments other than Saam acupuncture were well controlled in five cases.

In the case of a sleep disorder caused by a traffic accident, tonification of gall bladder improved sleep quality [15]. In traditional Chinese medicine (TCM), the gall bladder serves to buffer psychological anxiety. Because the cause of insomnia was determined to be a gallbladder deficiency, gallbladder tonification was selected as the solution.

Modified Saam acupuncture was used to treat 17 patients with refractory, sudden sensory-neuronal hearing loss of more than 3 weeks after a failed trial of conventional treatment including corticosteroids. The total improvement rate at 70.4 days after the initial visit was 47.1%. Thus, Saam acupuncture might be effective for refractory sensory-neuronal hearing loss in which conventional therapy has failed [16].

A 30-year-old woman with a right adnexal mass was treated with Saam acupuncture for 14 weeks. After treatment, transvaginal sonography revealed disappearance of the right adnexal mass. This effect may have been evoked by modification of autonomic nerve activity as a result, for example, of reflex alteration of ovarian sympathetic nerve activity [17].

Modified Saam acupuncture (LU8, BL66, SI5, TE4, and CV12) was used to treat 10 cancer patients for 2 weeks with 4 sessions. CD3+, CD8+, and T-cell subsets were significantly increased and the fatigue severity scale score was significantly decreased. Therefore, Saam acupuncture may improve the immune system [18].

Finally, peripheral capillary oxygen saturation (SpO_2_) was significantly increased in 18 amyotrophic lateral sclerosis patients with respiratory dysfunction who were treated with lung tonification [19].

### 4.2. Saam Acupuncture Clinical Research

Clinical studies of Saam acupuncture were mainly performed in relation to musculoskeletal pain and autonomic nervous system regulation (Table 4). Meridian identification was predominantly used when Saam acupuncture was applied to musculoskeletal pain. For example, when leg pain and numbness were present due to a herniated disc, bladder tonification was selected if the symptom occurred towards the back of the leg, and gall bladder tonification was used if the symptom occurred towards the lateral side of the leg, according to the flowing area of the meridian [20–22]. This principle was applied equally to knee pain derived from osteoarthritis [23], posterior ear pain due to Bell's palsy [24], and chronic tension headache [25]. Musculoskeletal pain-related clinical researches of Saam acupuncture mentioned above showed good results.

In clinical trials related to autonomic nervous system dysfunction, visceral pattern identification was used as the diagnostic criteria. For example, in case of Hwa-byung (Korean somatization disorder) [26–28], the heart or pericardium meridian, which is associated with psychological states in TCM, was selected. A tonification of pericardium was effective on the treatment of Hwa-byung. When the balance of the autonomic nervous system was disrupted by night-shift working [29], the gallbladder meridian that fits the psychological proportion was selected. A tonification of gallbladder could attenuate the imbalance between sympathetic and parasympathetic activities. When the face temperature dropped because of smoking [30], the fire acupoints of a fire organ (heart) and water organ (kidney) (HT8 and KI2, resp.) were selected as the treatment points. 5 of the 7 subjects showed increased temperatures after fire tonification treatment. In order to decrease hypertension in stroke patients [31], the bladder meridian, which has water and cold attributes, was applied. After 30 minutes of treatment, a tonification of the bladder meridian significantly depressed the systolic and diastolic blood pressure.

Although the method of setting the test and control groups differed slightly for each study, the use of a sham needle device for the control group appears to be the most desirable condition for scientific research. The Park sham needle was used to blind the participants [32], and the Kim sham needle was used to blind both the practitioner and participants [30].

Comparing the simultaneous application of Saam acupuncture and general body acupuncture and a single application of general body acupuncture is not suitable [20–22] because the amount of stimulation is different. Treating the control group by needling a nonacupoint is also problematic because the selection of nonacupoint near the five shu points could result in similar neurobiological activity. Notably, no significant difference was observed between the test and control groups in the Hwa-byung studies [26, 27]. In fatigue studies, the test group as well as the control group exhibited statistically significant effects in the multidimensional fatigue scale [33].

## 5. Neurobiological Mechanisms of Saam Acupuncture for Clinical Application

Saam acupuncture principally uses the acupoint below the elbow and knee joint. The five shu points occupy large areas in the cortical representation in the postcentral sensory gyrus in the brain. Cho et al. [34] stated that, based on their knowledge of Western medicine, it is difficult to believe that acupuncture treats organ-related disorders and diseases by direct control of organs. Acupuncture may first stimulate or activate the corresponding brain cortex via the central nervous system, thereby controlling chemical or hormone release to the diseased or disordered organs for treatment. From this point of view, inserting a needle in the distal limb may be more advantageous for inducing physiological activity caused by acupuncture than needling in the trunk.

Acupuncture likely has an effect on homeostasis via the somatic autonomic reflex [35]. This homeostasis between the sympathetic and parasympathetic nerve is frequently believed to be the scientific basis for the concept of the balance between yin and yang in TCM. The neural pathways involved in this acupuncture action have been well investigated in animal studies [36, 37].

Sato [37] showed that acupuncture stimulation in the extremity facilitates gastric motility through a somatic parasympathetic reflex associated with the vagal nerve in animal studies. In his studies, the response disappeared after either spinal transaction at the cervical level or bilateral severance of the vagal nerves. This suggests that a mechanism exists at the brain level related to a somatic parasympathetic reflex through the vagal nerve.

Nishijo et al. [38] revealed that acupuncture stimulation at PC4 decreased the heart rate as a result of the reciprocal coordination of an increase in cardiac vagal activity and a decrease in cardiac sympathetic activity in healthy humans. In this case, the reflex pathway might involve an afferent somatic nerve that goes to the brain and spreads to various structures in the hypothalamus, the midbrain, the medulla, and eventually to the autonomic efferent nerve.

Acupuncture stimulation on the abdomen impedes gastric motility [37]. Studies also showed that the response was maintained after spinal transection at the cervical level but was absent after bilateral severance of the gastric sympathetic nerve. This suggests that the reflex pathway is propagated within the spinal level, and acupuncture points within certain spinal segments in the trunk tend to affect the functioning of the organs that receive autonomic innervation from the same spinal segments through the sympathetic nerve.

The effect of acupuncture stimulation varies depending on the depth of the insertion, the respiratory phase, stimulation dose, and observation period. Mori et al. [39] showed that needling at TE5 induced pupillary constriction by increasing parasympathetic nerve action. An increase in parasympathetic nerve function was observed after gentle, superficial acupuncture stimulation. Bäcker et al. [40] revealed that, when inserting the needle at LI4, high-dose stimulation resulted in a greater increase in sympathetic nerve activity than low-dose stimulation.

Haker et al. showed that stimulation of the thenar muscle (LI11) resulted in a significant increase in sympathetic and the parasympathetic activity during the stimulation period and during the poststimulation period. A significant decrease in the heart rate at the end of the poststimulation period was also demonstrated. Superficial needle insertion into the skin overlaying the right thenar muscle caused a pronounced increase in the balance of both the sympathetic and parasympathetic activities during the 60 min poststimulation period [41].

Tanaka et al. demonstrated that SES (superficial needling exhalation phase in a sitting position) stimulation significantly decreased headache intensity, with a strong trend towards decreasing static electromyography (EMG) activity compared to CONT (without considering the respiratory phase) stimulation [42]. This suggests that the effect of acupuncture is derived from point selection of matching symptoms as well as consideration and utilization of the patient's respiratory phase during stimulation. The above finding suggests that, when applying Saam acupuncture for parasympathetic nerve activation or autonomic nervous system homeostasis, inserting a needle into the skin layer while considering the respiratory phase and stimulating the needle gently and lightly is the most effective.

In the neurobiological model, point specificity does not appear to exist with regard to musculoskeletal action, but five shu points, including PC5, PC6, and ST36, have some point specificity with regard to systemic action at the brain level. This acupuncture-point specificity has been attributed to the presence of a deep nerve [43]. However, other acupuncture points overlying the same deep nerve are not effective.

The Saam acupuncture method includes a variety of combinations of five shu points, and each combination is believed to have a distinct effect. However, the specificity of each Saam acupuncture treatment has not been yet identified from a neurobiological perspective. Therefore, when applying Saam acupuncture in the clinic as medical acupuncture, an approach that focuses on the balance and homeostasis of the autonomic nervous system appears to be desirable. Further, revealing the difference between the Yin meridian and Yang meridian treatments, which are anatomically divided into the medial and lateral sides of the body, is necessary from the viewpoint of the neurobiological mechanism.

## 6. Conclusion

The original Korean acupuncture method, Saam acupuncture, was invented 400 years ago by a Korean Buddhist monk based on the foundation of Dongeuibogam and Chimgoogyeong-heombang. He embodied Nan-jing's theory as an acupuncture treatment. Because Saam acupuncture was created on the basis of the theory of oriental medicine, approaching Saam acupuncture in the clinic via the oriental medical diagnosis is easy. However, for Saam acupuncture to be recognized as a medical acupuncture and to be used as a universal treatment in many countries, the mechanism of Saam acupuncture should be explained through scientific verification. Saam acupuncture, which operates with five shu points as a main aspect of treatment, has the advantage of increasing parasympathetic nerve activity and adjusting the balance of the autonomic nervous system. To maximize this effect, inserting a needle into the skin layer and providing gentle and light stimulation while considering the respiratory phase may be desirable.

## Figures and Tables

**Table 1:** Saam's combination of five shu points for deficiency and excess of the meridians.

Meridian	Deficiency				Excess			
	Tonify		Sedate		Tonify		Sedate	
Lung	SP3	LU9	HT8	LU10	HT8	LU19	KI10	LU5
Large intestine	ST36	LI11	SI5	LI5	SI5	LI5	BL66	LI2
Stomach	SI5	ST41	GB41	ST43	GB41	ST43	LI1	ST45
Spleen	HT8	SP2	LR1	SP1	LR1	SP1	LU8	SP5
Heart	LR1	HT9	KI19	HT3	KI19	HT3	SP3	HT7
Small intestine	GB41	SI3	BL66	SI2	BL66	SI2	ST36	SI8
Bladder	LI1	BL67	ST36	BL54	ST36	BL54	GB41	BL65
Kidney	LU8	KI7	SP3	KI3	SP3	KI3	LR1	KI1
Pericardium	LR1	PC9	KI10	PC3	KI10	PC3	SP3	PC7
Triple energizer	GB41	TE3	BL66	TE2	BL66	TE2	ST36	TE10
Gall bladder	BL66	GB43	LI1	GB44	LI1	GB44	SI5	GB38
Liver	KI10	LR8	LU8	LR4	LU8	LR4	HT8	LR2

**Table 2:** Saam's combination of five shu points for cold and heat symptoms of the meridians.

Meridians	Cold				Fire			
	Tonify		Sedate		Tonify		Sedate	
Lung	HT8	LU10	LU5	KI10	LU5	KI10	SP3	LU9
Large intestine	SI5	ST41	LI2	BL66	LI2	BL66	SI5	ST41
Stomach	ST41	SI5	ST44	BL66	ST44	BL66	ST36	BL54
Spleen	SP2	HT8	SP9	KI10	SP9	KI10	SP3	KI3
Heart	HT8	KI2	HT3	KI10	HT3	KI10	HT8	KI2
Small intestine	SI5	BL60	SI2	BL66	SI2	BL66	SI8	ST36
Bladder	SI5	BL60	SI2	BL66	SI2	BL66	ST36	BL54
Kidney	HT8	KI2	KI10	HT3	KI10	HT3	SP3	KI3
Pericardium	HT8	PC8	PC3	HT3	PC3	HT3	SP3	PC7
Triple energizer	TE6	BL60	TE2	BL66	TE2	BL66	TE6	BL60
Gall bladder	GB38	SI5	GB43	BL66	GB43	BL66	BL54	GB34
Liver	LR2	HT8	KI10	LR8	KI10	LR8	LR3	SP3

**Table 3:** Saam acupuncture clinical case reports.

Year	Disease	*N*	Period
1964	Eyelid edema	1	7 days
1975	Indigestion, HNP of lumbar	3	2–5 days
1981	Neurosis, duodenal ulcer	2	14 days
1998	Hyperemesis gravidarum	1	15 days
2002	Hwa-byung	2	5 months
2002	Wei symptom	1	2 months
2002	Oral dyskinesia	1	1 month
2003	Hemichorea-hemiballism	1	8 days
2003	Sequelae of CVA	1	10 months
2003	Hemorrhoid	2	10 days
2003	Fracture	1	1 month
2004	Lumbar compression fracture	1	14 days
2004	Insomnia	20	3 days
2006	Otitis media	3	1 month
2007	Tic disorder	1	3 months
2008	Knee strain	1	1 week
2008	Inflammatory acne	1	2 months
2009	Cancer pain	1	2 months
2010	Hearing loss	17	10 weeks
2011	Foot coldness	1	1 week
2012	ALS	1	5 days
2012	Meniere's disease	1	3 weeks
2013	Adnexal mass	1	14 weeks
2013	Calcific tendinitis of shoulder	1	7 days
2013	ALS	18	5 days
2014	Cancer	10	14 days
2014	Chronic post-stroke hemiparesis	7	1 month

*N*: number, ALS: amyotrophic lateral sclerosis, CVA: cerebrovascular accident, and HNP: herniated nucleus pulposus.

**Table 4:** Saam acupuncture clinical research.

Year	Disease	*N*	Period	Method
1999	Lumbar HNP	28	5 weeks	SA + GBA/GBA
2002	Sciatica and back pain	40	10 days	SA + GBA/GBA
2002	Lumbar HNP	29	1–10 weeks	SA + GBA/GBA
2003	Dysarthria	20	10 days	SA/GBA
2004	Hypertension	60	1 hour	SA/BR
2004	Posterior ear pain	30	10 days	SA/GBA
2006	OA of knee	78	4 weeks	SA/ShA (Park sham needle)
2007	Chronic tension headache	26	2 weeks	SA/ShA (nonacupoint)
2007	Dysmenorrhea	8	1 month	SA/ShA (acupoint not related to gynecological disorders)
2007	Obesity	60	4 weeks	SA/ShA (Kim sham needle)/NA
2007	Fatigue	56	4 weeks	SA/ShA (nonacupoint)
2007	Hwa-byung	23	2 weeks	SA/ShA (nonacupoint)
2008	Hwa-byung	52	2 weeks	SA/ShA (nonacupoint)
2011	Autonomic nerve disorder	6	6 days	SA/ShA (Park sham needle)
2011	Hwa-byung	50	2 weeks	SA/ShA (nonacupoint)
2012	Facial temperature	7	6 days	SA/ShA (Kim sham needle)
2013	Peripheral neuropathy	10	8 weeks	SA/NA

HNP: herniated nucleus pulposus; OA: osteoarthritis, SA: Saam acupuncture; GBA: general body acupuncture; ShA: sham acupuncture; BR: bed rest; NA: no acupuncture; *N*: number.
